# Enhanced Microscopic Dynamics of a Liver Lipid Membrane in the Presence of an Ionic Liquid

**DOI:** 10.3389/fchem.2020.577508

**Published:** 2020-11-19

**Authors:** Veerendra K. Sharma, Sajal K. Ghosh, Victoria García Sakai, R. Mukhopadhyay

**Affiliations:** ^1^Solid State Physics Division, Bhabha Atomic Research Centre, Mumbai, India; ^2^Homi Bhabha National Institute, Mumbai, India; ^3^Department of Physics, School of Natural Sciences, Shiv Nadar University, Greater Noida, India; ^4^Rutherford Appleton Laboratory, ISIS Pulsed Neutron and Muon Facility, Science and Technology Facilities Council, Didcot, United Kingdom

**Keywords:** ionic liquids, lipid membrane, neutron scattering, lateral motion, internal motion

## Abstract

Ionic liquids (ILs) are an important class of emerging compounds, owing to their widespread industrial applications in high-performance lubricants for food and cellulose processing, despite their toxicity to living organisms. It is believed that this toxicity is related to their actions on the cellular membrane. Hence, it is vital to understand the interaction of ILs with cell membranes. Here, we report on the effects of an imidazolium-based IL, 1-decyl-3-methylimidazolium tetrafluoroborate (DMIM[BF4]), on the microscopic dynamics of a membrane formed by liver extract lipid, using quasielastic neutron scattering (QENS). The presence of significant quasielastic broadening indicates that stochastic molecular motions of the lipids are active in the system. Two distinct molecular motions, (i) lateral motion of the lipid within the membrane leaflet and (ii) localized internal motions of the lipid, are found to contribute to the QENS broadening. While the lateral motion could be described assuming continuous diffusion, the internal motion is explained on the basis of localized translational diffusion. Incorporation of the IL into the liver lipid membrane is found to enhance the membrane dynamics by accelerating both lateral and internal motions of the lipids. This indicates that the IL induces disorder in the membrane and enhances the fluidity of lipids. This could be explained on the basis of its location in the lipid membrane. Results are compared with various other additives and we provide an indication of a possible correlation between the effects of guest molecules on the dynamics of the membrane and its location within the membrane.

## Introduction

Ionic liquids (ILs) are a particular class of organic salts in which the ions are poorly coordinated, which results in low melting temperatures (Hayes et al., 2015; Egorova et al., 2017). ILs are non-explosive, non-flammable and have good thermal stability and high ionic conductivity (Hayes et al., 2015; Egorova et al., 2017). These ILs have various widespread industrial applications, such as in high-performance lubricants, in chemical and polymer synthesis, in energy harvesting, and in food and cellulose processing (Plechkova and Seddon, 2008; Plechkova et al., 2009; Hayes et al., 2015; Egorova et al., 2017). However, recent studies (Matzke et al., 2007; Jeong et al., 2012; Liang et al., 2013; Bakshi et al., 2020) have suggested that ILs are toxic to various organisms. It has been shown that toxicity strongly depends on the lipophilicity of the IL, generally increasing with IL chain length (Matzke et al., 2007; Jeong et al., 2012; Liang et al., 2013). The origin of the toxicity of the IL is mainly attributed to their interaction with the cellular membrane (Jeong et al., 2012; Benedetto and Ballone, 2016; Bakshi et al., 2020), which provides the interface between an organelle or a cell, and the surrounding external environment. It is a heterogeneous mixture of various lipids, proteins, and other small molecules. The main constituents of the cell membrane are the lipids and thus a lipid membrane can be used as a model cell membrane system.

**Figure 1:**
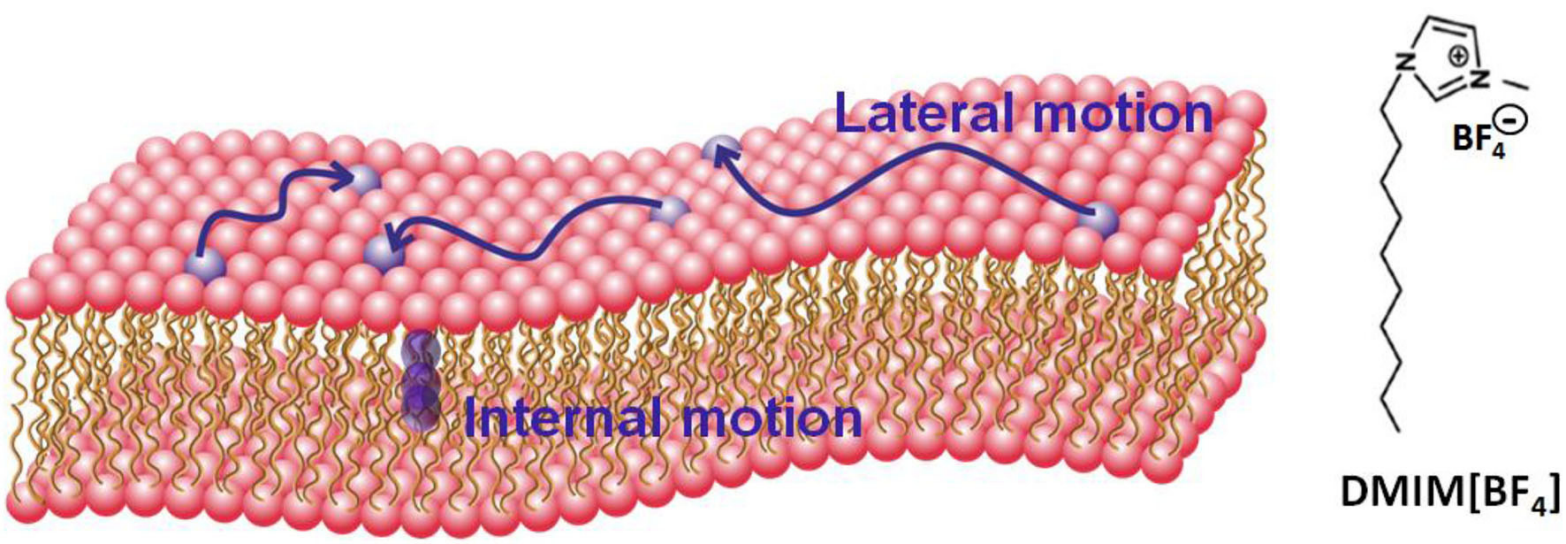
Schematic of a lipid bilayer and molecular configuration of the ionic liquid DMIM[BF4]. Two distinct motions (i) lateral motion within the leaflet and (ii) localized internal motion in the lipids are also shown.

Imidazolium-based cations are a popular class of ILs which have shown high levels of toxicity against several classes of organisms (Pendleton and Gilmore, 2015). Recently (Bakshi et al., 2020), we have studied the effects of various imidazolium-based ILs [C_n_MIM]^+^[BF4] (*n* varies from 2 to 10) on different cancerous cells such as human breast, colon and liver cells. Our work showed that 1-decyl-3-methylimidazolium tetrafluoroborate [C_10_MIM]^+^[BF4] [or [DMIM][BF4]] has the highest toxicity toward liver cancerous cells, which we suggest is due to the modulation of the structure and dynamics of the liver membrane due to the incorporation of IL (Bakshi et al., 2020). In the present study, we investigate this possible modulation in more detail, specifically on the effect of [DMIM][BF4] on the dynamics of a membrane from liver extract lipid. The structure of the IL is shown in Figure 1. We choose this as our model system instead of a simpler more commonly used single lipid model membrane, since it more physiologically relevant. The liver lipid extract membrane contains the commonly found lipids in all eukaryotic cells, namely a mixture of lipids including phosphatidylcholine (PC), phosphatidylethanolamine (PE), phosphatidylinositol (PI), and cholesterol.

Membrane dynamics play a key role in the viscoelastic properties of the cell membrane, which are important in various physiological processes such as cell signaling, protein-protein interaction, etc. Lipid membranes exhibit a complex hierarchical dynamical behavior owing to multiple relaxation processes on the local and global scales (Tocanne et al., 1994; Lipowsky and Sackmann, 1995; Marquardt et al., 2017; Nagao et al., 2017; Sharma et al., 2019a,b). For example, at a local scale, an individual lipid molecule performs distinct motions such as vibrations, conformational changes, protrusions, molecular rotations, lateral motions within the leaflet, flip flop motions from one leaflet to another, etc. On the other hand, at a larger and more global scale, the whole lipid bilayer may undergo bending motions, thickness fluctuations, shape fluctuations, etc. These motions occur in a wide range of time scales ranging from vibrations at ~fs to flip flop motions which take a few hours, and over a wide variety of length scales, from molecular rotations at a few Ås to the macroscopic deformation of the vesicles at the micrometer scale. To investigate these motions, various experimental methods have been used, such as dynamic light scattering (Hassan et al., 2015; Sharma et al., 2020), fluorescence spectroscopy (Machán and Hof, 2010; Singh et al., 2019), electron paramagnetic resonance (McConnell and Kornberg, 1971), nuclear magnetic resonance (Perlo et al., 2011), quasielastic neutron scattering (Busch et al., 2010; Armstrong et al., 2011; Sharma et al., 2015a, 2017a,b; Dubey et al., 2018; Sharma and Mukhopadhyay, 2018), neutron spin echo (Boggara et al., 2010; Lee et al., 2010; Woodka et al., 2012; Nickels et al., 2015). Each method has limited accessible temporal and spatial regimes. For example, dynamic light scattering is more suitable to study motions at time scales of the order of microseconds while quasielastic neutron scattering (QENS) is suitable to study dynamics from nanoseconds to sub-picoseconds and length scales from Angstroms to a few nanometers (Gardner et al., 2020). Furthermore, neutron spin echo (NSE) is more suitable to prove the relatively slower motions, up to 0.1 μs, taking place at large spatial scales, up to 0.1 μm (Gardner et al., 2020). It is therefore evident that to obtain the detailed dynamical landscape one needs to combine results from different experimental methods.

Here, we report the effects of [DMIM][BF4] IL on the microscopic dynamics of liver lipid membrane studied using QENS techniques. Two distinct motions, (i) lateral motion of the lipid within the leaflet and (ii) localized internal motions of the lipid are observed (A schematic of a lipid bilayer and these motions are shown in Figure 1). Our measurements suggest that the incorporation of the IL in the membrane modulates both of these dynamical processes. We note that the dynamics of ILs have also been investigated using the QENS technique (Triolo et al., 2006; Mamontov et al., 2009; Aoun et al., 2010; Burankova et al., 2018; Nemoto et al., 2018), however in this work we focus on the dynamics of the lipids and how they are affected by the presence of IL, and not on the dynamics of the IL themselves, since the concentration is small (see experimental details).

## Experimental Methods

### Preparation of Unilamellar Vesicles

Liver lipid extract (bovine) solution in chloroform was procured from Avanti Polar Lipids (USA). This extract is a mixture of various lipids PC:PE:PI:lysoPI:Cholesterol:other lipids = 42:26:9:1:5:17 (weight%). The ionic liquid [DMIM][BF4], and D_2_O (99.9%) were obtained from Sigma Aldrich (USA). Deuterated water was used in order to minimize the scattering contribution from the solvent, since the neutron scattering cross-section of deuterium is lower than hydrogen by more than an order of magnitude (σ_D_ << σ_H_). Large unilamellar vesicles (LUV) composed of liver lipids were prepared using the extrusion method (Sharma et al., 2015b, 2016a,b; Mitra et al., 2020). Briefly, organic solvent was evaporated to obtain lipid films. These films were hydrated by D_2_O which followed 3 freeze-thaw cycles by keeping the lipid suspension in a warm water bath (50°C) and in a freezer (−80°C). The suspension was extruded 21 times through a 100 nm pore sized polycarbonate filter using a mini extruder from Avanti Polar Lipids Inc. Two different samples, 5% (*w/w*) liver lipid LUV, and 5% (*w/w*) liver lipid LUV with 10 wt% [DMIM][BF4] (with respect to liver lipid) were used. It should be noted that the main contribution to the quasielastic scattering signal comes from the liver lipid and that only ~7% is from the DMIM IL, making it small enough to be neglected from the analysis.

### Quasi-Elastic Neutron Scattering (QENS) Experiment

QENS experiments were carried out on the liver lipid-based LUVs with and without [DMIM][BF4], using the time-of-flight neutron backscattering spectrometer, IRIS (Carlile and Adams, 1992) at ISIS facility, UK. To minimize multiple scattering effects, both samples were filled into annular aluminum sample holders with an annular spacing of 0.5 mm to ensure a sample transmission of more than 90%. The IRIS spectrometer was used with the PG(002) analyzer giving an energy resolution of ΔE = 17 μeV (full width at half-maximum), and with a detector coverage spanning a wave vector transfer range of 0.5–1.8 Å^−1^. In the offset mode, the spectrometer provided an energy transfer range of −0.3 to +1.0 meV. To estimate the solvent contribution, QENS measurements were also carried out on pure D_2_O. Measurements were carried out at two distinct temperatures, 37°C (physiological temperature) and 57°C. A QENS measurement was also carried out on vanadium, a good elastic incoherent scatterer, to obtain the instrument resolution. Standard data reduction was carried out using MANTID software (Taylor et al., 2012).

## Results and Discussion

In a quasielastic neutron scattering experiment, the scattered intensity is proportional to the double scattering cross section, which gives the probability of scattered neutrons within the solid angle element d**Ω**, about the direction **Ω** and with an energy exchange of *dE* = *E*_f_-*E*_i_ (*E*_f_ and *E*_r_ are the final and initial energy of the neutron, respectively). The double scattering cross section can be written as Bee (1988)

(1) $$\begin{array}{l} \frac{d^{2}\sigma }{dEd\Omega }\propto \frac{k_{f}}{k_{i}}\left[\sigma_{coh}S_{coh}\left(Q,E\right)\;+\;\sigma_{inc}S_{inc}\left(Q,E\right)\right] \end{array}$$

where, *S*_*coh*_ and *S*_*inc*_ are the coherent and incoherent scattering laws, and σ_*coh*_ and σ_*inc*_ are the coherent and incoherent scattering cross sections; and ***Q*** is the momentum transfer in the scattering process. In the case of hydrogenous samples, the scattered intensity is dominated by incoherent scattering from the hydrogen atoms in the sample, which is due to their exceptionally high incoherent scattering cross section compared to the coherent or incoherent scattering cross-section of any other atoms ($\sigma_{inc}^{H}$>> $\sigma_{inc/coh}^{any\;atom}$). In this case, Equation (1) can be written as

(2) $$\begin{array}{l} \frac{d^{2}\sigma }{dEd\Omega }\propto \frac{k_{f}}{k_{i}}\sigma_{inc}S_{inc}\left(Q,E\right) \end{array}$$

The incoherent scattering law *S*_*inc*_ (*Q*,ω) is the double Fourier transform of *G*_*s*_(*r, t*) which provides the probability of finding a particle at position *r* and time t provided the same particle was at position *r* = 0 at *t* = 0. Hence, a QENS experiment from a hydrogenous system provides information about the self-diffusion or single particle motion of the protons in the system. Since our interest lies in the study of the dynamics of the liver lipid membrane, D_2_O was used as a solvent while preparing the LUVs to minimize the solvent contribution. The contribution to the measured scattering signal from the dynamics of the lipid liver was extracted by subtracting the weighted D_2_O signal from the solution data (Sharma et al., 2015b, 2016b).

**Figure 2:**
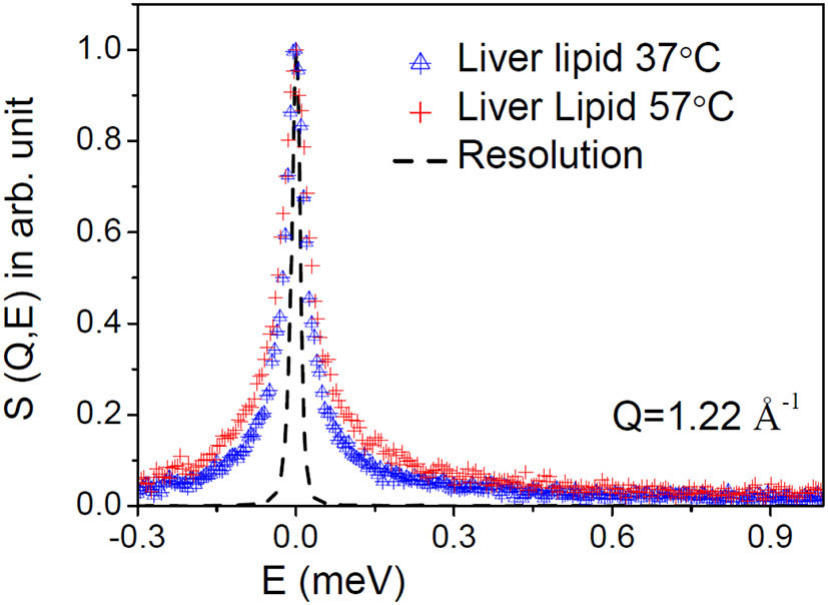
Typical measured QENS spectra at a representative *Q* = 1.22 Å^−1^ for the liver lipid membrane at 37 and 57°C. The evolution of the dynamics at higher temperature is evident. The contribution of the solvent (D_2_O) has been subtracted, and the resultant spectra are plotted normalized to the peak amplitudes. Instrument resolution is shown by a dashed line.

D_2_O subtracted QENS spectra for the liver lipid membrane at both the temperatures are shown in Figure 2 at a typical *Q*-value of 1.22 Å^−1^. The instrument resolution, as measured using a standard vanadium sample, is also shown in the figure. For direct comparison, spectra are normalized to 1 by dividing the respective spectra by the peak value, S(*Q*,0). Significant quasielastic (QE) broadening is observed for the liver lipid membrane at both temperatures, indicating the presence of stochastic molecular motions of the lipid in the temporal window accessible by the IRIS spectrometer.

**Figure 3:**
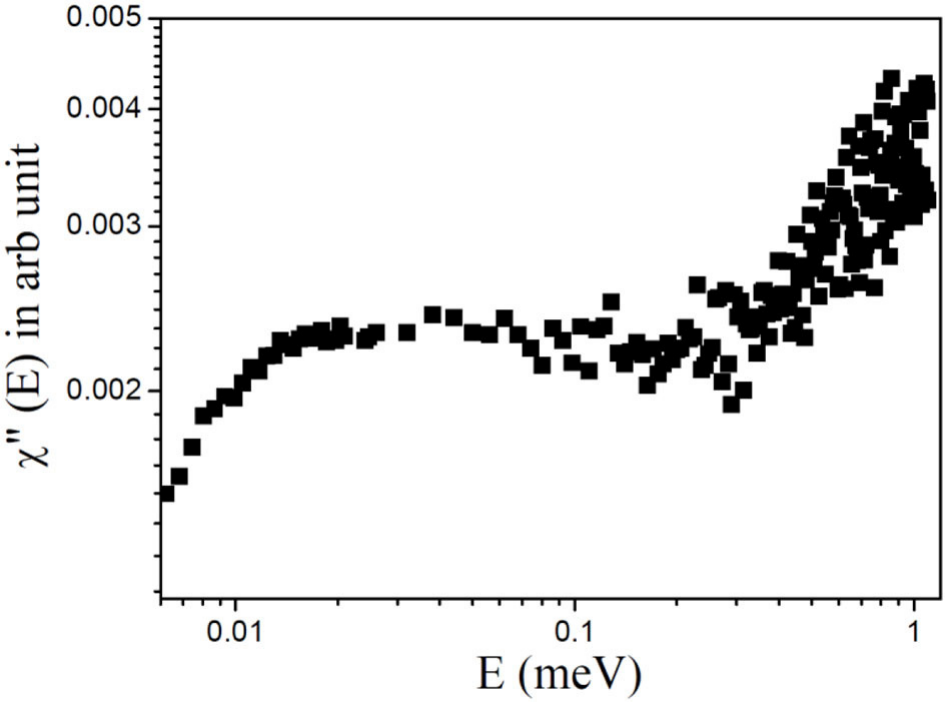
Dynamic susceptibility, χ″(E), for the liver lipid membrane at 37°C.

Plotting the data in terms of the dynamic susceptibility is a useful way to find out the number of relaxation processes without employing any detailed data analysis. This approach is possible since a relaxation process with a characteristic time (τ) shows a peak in the dynamic susceptibility at energy transfer *E* = ℏ*/*τ, where ℏ is the reduced Planck's constant. The scattering data are converted into the imaginary component of the dynamic susceptibility, χ″(*E*), by dividing the *Q*-averaged intensity, *S*(*E*), by the Bose population factor, *n*_B_(E) ≈ *k*_*B*_*T/E* (under the approximation that *E* << *k*_*B*_*T*), where *k*_*B*_ is the Boltzmann's constant and *E* = ℏω is the energy transfer. The resulting profile of χ″(*E*) vs. *E* for liver lipid membrane obtained at 37°C is shown in Figure 3. It can be inferred from the susceptibility spectra that the dynamics of the liver lipid membrane cannot be viewed as a single relaxation process. The existence of a minimum in the susceptibility spectra indicates the presence of two relaxation processes.

As mentioned before, molecular motions in the time scale from nanoseconds to picoseconds and length scales from Angstroms to few nanometers can be probed by QENS. On these length and time scales, two different motions which can contribute to the observed data are: (i) the lateral motion of lipid molecules within the leaflet and (ii) relatively faster internal motions (Sharma et al., 2015b, 2016a,b; Mitra et al., 2020). Assuming both motions are independent of each other, the scattering law for the lipid membrane can be written as

(3) $$\begin{array}{l} S_{mem}\left(Q,E\right)=S_{lat}\left(Q,E\right)⊗S_{\mathrm{int}}\left(Q,E\right) \end{array}$$

where, *S*_*lat*_(*Q,E*) and *S*_*int*_(*Q,E*) are the scattering functions corresponding to the lateral and internal motions, respectively. The nature of the lateral motion of the lipid in the membrane is debatable as a number of different models have been proposed, such as continuous diffusion (Armstrong et al., 2011), ballistic flow-like motions (Busch et al., 2010), localized diffusion (Wanderlingh et al., 2014), sub-diffusive motion (Flenner et al., 2009; Srinivasan et al., 2018), etc. We have assumed the simple continuous diffusion model, based on the recent study (Armstrong et al., 2011), which has shown that this is the case for the temporal and spatial scales accessible by QENS and valid at least, for distances greater than a lipid molecule diameter. The scattering law corresponding to the lateral motion is then expressed as:

(4) $$\begin{array}{l} S_{lat}\left(Q,E\right)=L_{lat}\left(\Gamma_{lat},E\right) \end{array}$$

where *L*_*lat*_*(*Γ_*lat*_*, E)* is a Lorentzian function, corresponding to the lateral motion of the lipid, and Γ_*lat*_ is its HWHM, which is inversely proportional to the timescale of the motion.

The internal motions of the lipid molecules correspond to motions that are spatially restricted by the chemical structure of the lipid molecule. Thus, after a relatively long relaxation time, there is a finite probability of finding the scattering center within a given molecular volume. This leads to an elastic component in the scattering law and we can write the scattering law for the internal motions as,

(5) $$\begin{array}{l} S_{\text{int}}\left(Q,E\right)=A\left(Q\right)\delta \left(E\right)\;+\;\left(1-A\left(Q\right)\right)L_{\text{int}}\left(\Gamma_{\text{int}},E\right) \end{array}$$

The first term represents the elastic part where *A*(*Q*) is the elastic incoherent structure factor (EISF). The second term corresponds to the quasielastic part which is characterized by a single Lorentzian *L*_*int*_(Γ_*int*_,E) function with Γ_*int*_ is its HWHM.

Combining Equations (4) and (5), the resultant scattering law (Equation 3) can be written as

(6) $$\begin{array}{l} S_{mem}\left(Q,E\right)=[A\left(Q\right)L_{lat}\left(\Gamma_{lat},E\right)+\left(1-A\left(Q\right)\right)L_{tot}(\Gamma_{lat}\\ \;+\Gamma_{\mathrm{int}},E)] \end{array}$$

**Figure 4:**
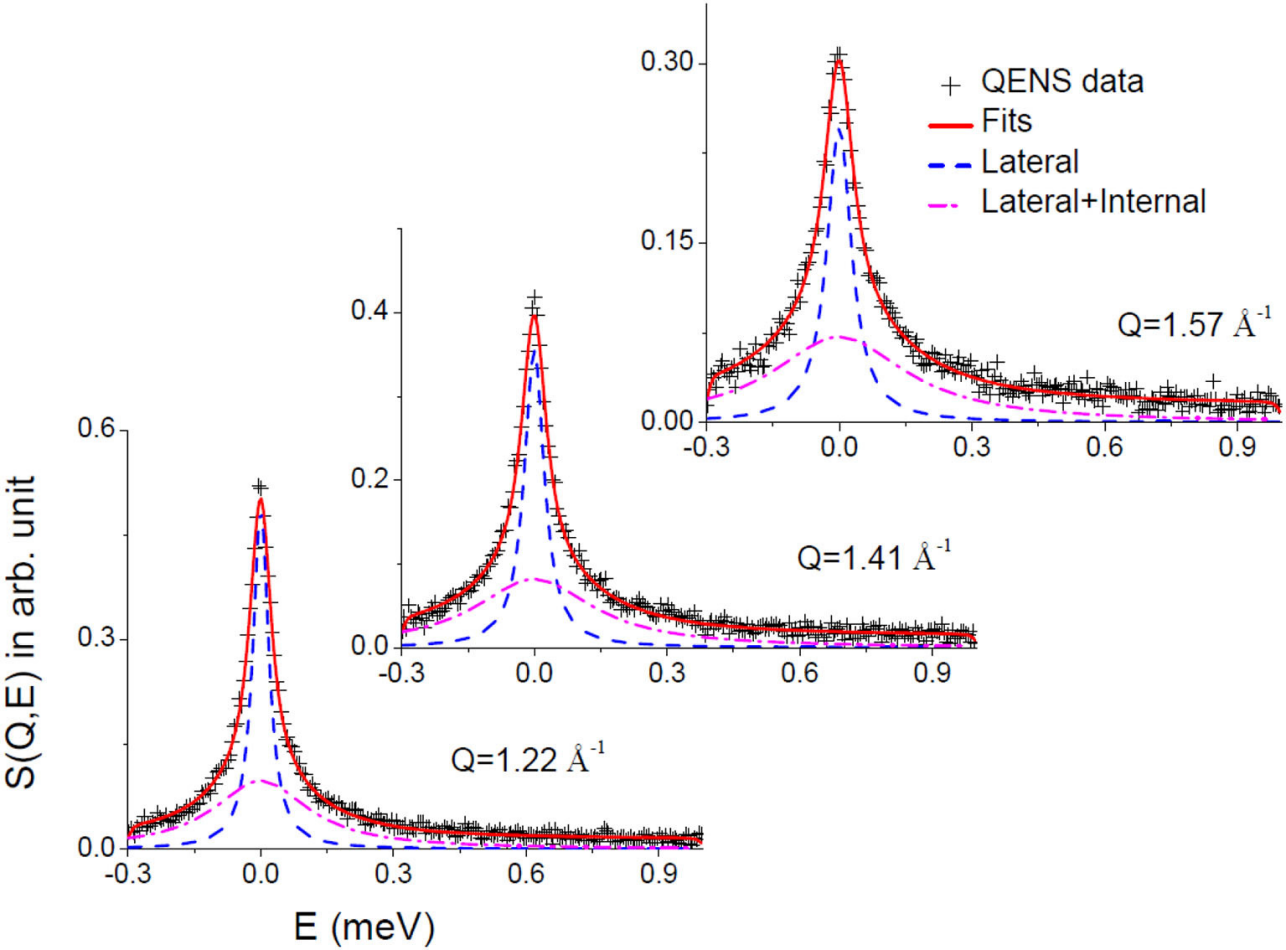
Typical fitted QENS spectra for liver lipid membrane at 57°C. Individual components corresponding to lateral and lateral with internal motions are also shown.

Convoluting Equation (6) with the resolution function (obtained by measuring the data from a standard vanadium sample), the parameters *A*(*Q*), Γ_lat_, and Γ_int_ were determined by least squares fit of the measured spectra. DAVE software (Azuah et al., 2009) developed at the NIST Center for Neutron Research has been used to analyze the QENS data. It is found that the scattering law given in Equation (6) describes the observed QENS data quite well for liver lipid membrane in the absence and presence of the IL for the entire *Q* range at both temperatures. Typical fitted QENS spectra for liver lipid membrane at 57°C at different *Q* values are shown in Figure 4. To gain more insight into the different dynamical processes, the fit parameters are used to obtain some more dynamical characteristic parameters for each of the processes (such as diffusion coefficients and extent of mobility).

### Lateral Motion

**Figure 5:**
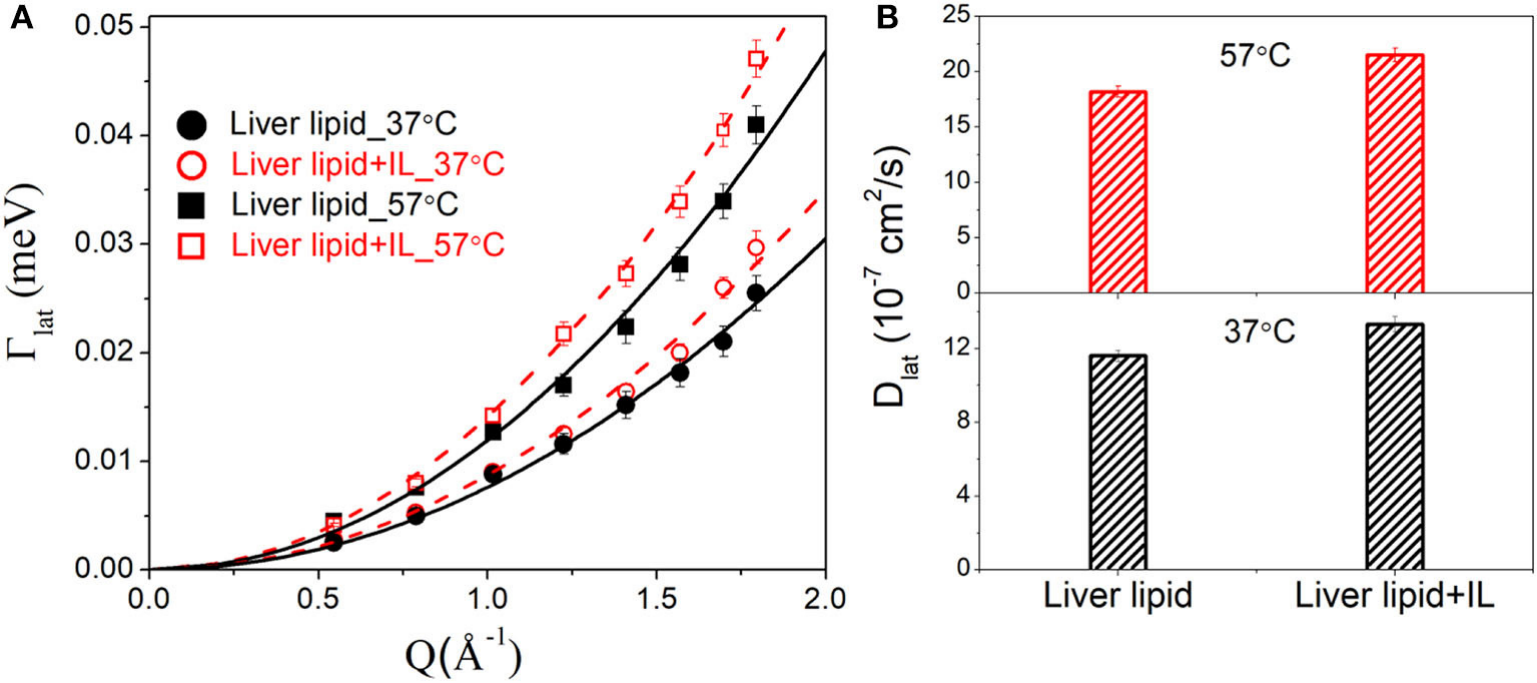
**(A)** Variation of HWHM's of Lorentzian corresponding to the lateral motion of liver lipid in the absence and presence of [DMIM][BF4] IL at 37 and 57°C. The solid and dashed lines the fits assuming Fick's law of diffusion. **(B)** Lateral diffusion coefficient *D*_lat_ for liver lipids in absence and presence of [DMIM][BF4] IL at 37 and 57°C.

The lateral motion of the lipid molecules within the leaflet is of principal interest since it plays a key role in various physiologically relevant membrane processes, such as cell signaling, membrane trafficking, location and activity of membrane proteins, cell recognition, etc. HWHM's of the Lorentzian corresponding to the lateral motion, Γ_lat_ for the liver lipid with and without [DMIM][BF4], at 37 and 57°C, are shown in Figure 5A. At both temperatures, addition of the IL increases the values of HWHM, indicating an enhancement in lateral diffusion. It is evident that for liver lipid in the absence and presence of [DMIM][BF4], Γ_lat_ increases quadratically with *Q* at both temperatures, passing through the origin, which indicates that the lateral motion of the lipid molecules can be described as a continuous diffusion, described by Fick's law $\Gamma_{lat}=D_{lat}Q^{2}$. A least squares fitting method is used to determine the lateral diffusion coefficient, *D*_*lat*_, for liver lipid with and without [DMIM][BF4] at both temperatures. Figure 5B shows that the incorporation of the IL accelerates the lateral motion of the lipids at both temperatures by about 15–20%. At the physiological temperature of 37°C, *D*_lat_ is found to be 11.6(±0.3) × 10^−7^ cm^2^/s which increases to 13.3(±0.4) × 10^−7^ cm^2^/s due to the incorporation of [DMIM][BF4]. At 57°C, D_lat_ is found to be 18.2(±0.5) × 10^−7^ cm^2^/s which increases to 21.5(±0.6) × 10^−7^ cm^2^/s.

### Internal Motion

**Figure 6:**
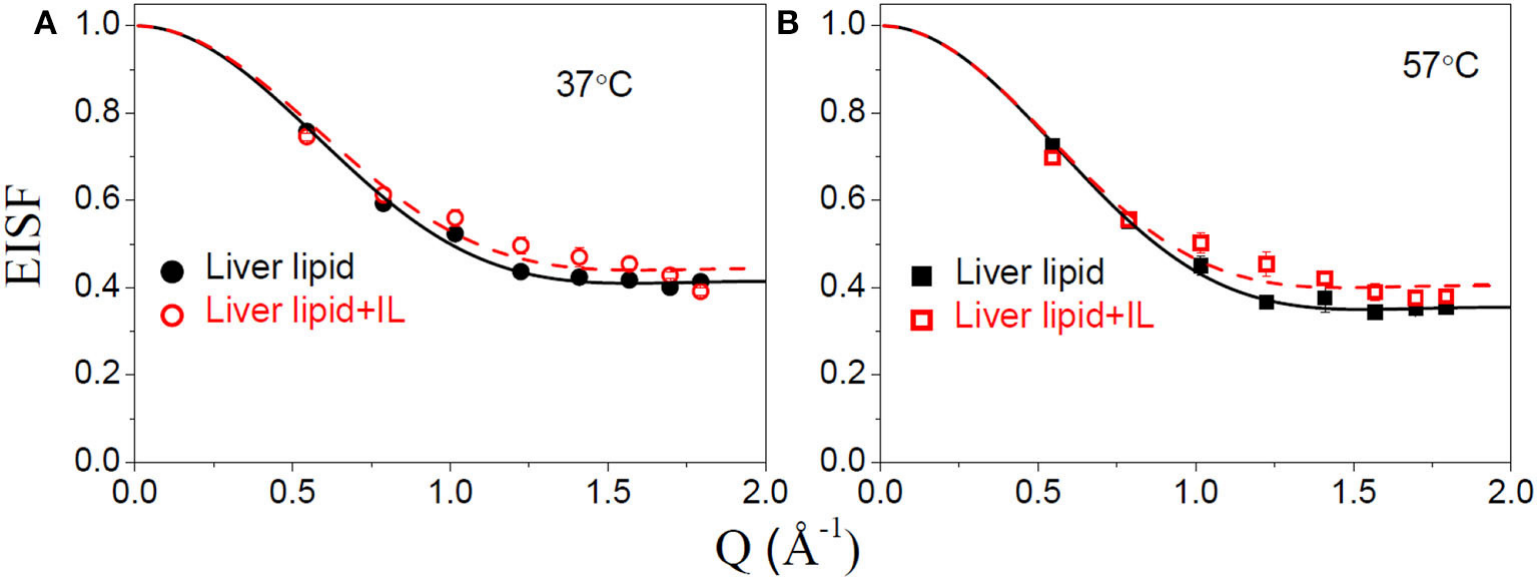
Elastic incoherent structure factor (EISF) corresponding to the internal motion of liver lipid in the pristine membrane and membrane with IL at **(A)** 37°C and **(B)** 57°C. The solid and dashed lines correspond to the fit assuming localized translational diffusion model as discussed in the text.

**Figure 7:**
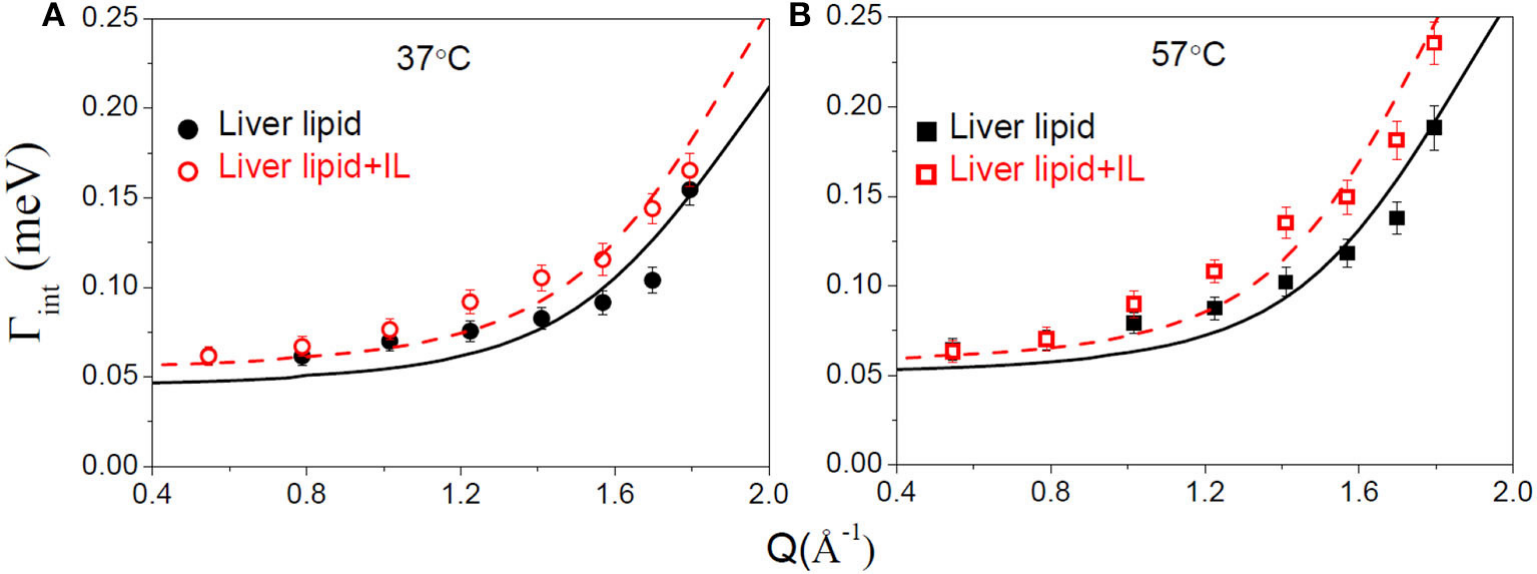
Variation of the HWHM, Γ_int_, corresponding to internal motions of liver lipid in the pristine membrane and membrane with IL at **(A)** 37°C and **(B)** 57°C. The solid and dashed lines correspond to the fits as per the localized translational diffusion model as discussed in the text.

The internal motion of the lipid is characterized by two parameters, the EISF and the HWHM, Γ_int_. Figures 6A,B show the EISFs for the liver lipids in the absence and presence of [DMIM][BF4] at 37 and 57°C, respectively. The internal motions of liver lipids are expected to be a complex mixture of motion, including reorientation of lipids, conformational motions, large amplitude oscillations, etc. The variation of Γ_int_ with *Q*, as shown in Figure 7, is very different to that observed for Γ_lat_. It may be noted that at low *Q*, Γ_int_ is nearly constant and approaches a non-zero value as *Q* tends to zero. At higher *Q*, Γ_*i*__nt_ increases and follows *Q*^2^ variation. This is a signature of localized translational diffusion within a spherical volume. We have successfully described the internal motions in similar systems (Sharma et al., 2010, 2015a; Dubey et al., 2018) by a model where all these various motions have been effectively taken into account. In this model, it is assumed that the hydrogen atoms belonging to lipids undergo localized translation diffusion (LTD) within a confined volume (Sharma et al., 2015a; Dubey et al., 2018). At lower *Q* (*QR* < π; where *R* is radius of confining spherical domain), that is when larger distances are probed, the behavior of Γ_int_ is independent of *Q*. At this length of scale, hydrogen atoms in the lipid molecules perform localized motions. Therefore, the behavior of Γ_int_ in this *Q* range is similar to that of rotational motions. However, at larger *Q* values (*QR* > π), where small distances are probed within the spherical domain, the usual *DQ*^2^ behavior corresponding to translational motion in an infinite medium is observed. It is likely that at a given temperature, all the hydrogen atoms belonging to a lipid molecule are not dynamically active. Assuming that on average only a fraction of hydrogen atoms are participating in the internal motions, the EISF can be written as Volino and Dianoux (1980)

(7) $$\begin{array}{l} A\left(Q\right)=p_{x}+\left(1-p_{x}\right){\left[\frac{3j_{1}\left(QR\right)}{QR}\right]}^{2} \end{array}$$

where *p*_*x*_ is the fraction of hydrogen atoms that are immobile on the observation time scale, *R* is the radius of the spherical domain and *j*_1_ is the first-order spherical Bessel function. It was found that the experimentally observed EISF could be described very well using Equation (7) for the liver lipid membranes with and without [DMIM][BF4] at both temperatures, as shown by the solid lines in Figures 6A,B. The obtained fitting parameters, *p*_x_ and *R* are given in Table 1. At 37°C, values of *p*_x_ = 0.41 and *R* = 2.8 Å indicate that on average about 59% of the hydrogen atoms participate in the localized translational diffusion, within a sphere with a radius of 2.8 Å. The size of the confining domain is correlated with the bilayer structure, more specifically with the area per lipid molecule and found to be consistent with studies on other lipid membranes (Busch et al., 2010; Mitra et al., 2020). Due to the addition of the IL, the fraction of immobile hydrogen atoms increases very slightly, but the radius of the spherical domain remains more or less unchanged. It is evident that addition of DMIM[BF4] does not affect the nature of the internal dynamics.

**Table 1:** Fraction of immobile hydrogen atoms (*p*_*x*_), radius of the spherical domain (*R*) and diffusivity correspond to the internal motion of the liver lipids in the membrane in the absence and presence of [DMIM][BF4].

***T* (°C)**	**Liver lipids**			**Liver lipids + [DMIM][BF4]**		
	***p_***x***_* (%)**	***R* (Å)**	***D_***int***_* (× 10^**−6**^ cm^**2**^/s)**	***p_***x***_***	***R* (Å)**	***D_***int***_* (× 10^**−6**^ cm^**2**^/s)**
37	41 (3)	2.8 (2)	12.3 (4)	43 (3)	2.8 (2)	14.9 (4)
57	35 (2)	2.9 (2)	15.0 (5)	39 (3)	3.0 (3)	18.4 (6)

For any model to be considered robust it should simultaneously describe both EISF and HWHM. The scattering law for the localized translational diffusion model as described above can be written as Volino and Dianoux (1980):

(8) $$\begin{array}{l} S_{\mathrm{int}}\left(Q,E\right)=\left[p_{x}+\left(1-p_{x}\right){\left[\frac{3j_{1}\left(QR\right)}{QR}\right]}^{2}\right]\delta \left(\omega \right)\\ \;+\left(1-p_{x}\right)[\frac{1}{\pi }\underset{\left\{l,n\right\}\neq \left\{0,0\right\}}{\sum }(2l\\ \;+1)A_{n}^{l}\left(QR\right)\frac{\hbar {\left(x_{n}^{l}\right)}^{2}D_{\mathrm{int}}/R^{2}}{{\left[\hbar {\left(x_{n}^{l}\right)}^{2}D_{\mathrm{int}}/R^{2}\right]}^{2}+E^{2}}] \end{array}$$

Where $A_{n}^{l}\left(QR\right)$; (*n, l* ≠ 0,0) is the quasielastic structure factor and values of it for different *n* and *l* can be calculated by using the values of $x_{n}^{l}$ which are given in Volino and Dianoux (1980) and are obtained by the numerical solution of the equations as described therein. Here, *D*_*int*_ is the diffusion coefficient for internal motion. Equation (8) involves an infinite sum of the Lorentzian whose widths are independent of *Q*, but whose weight factors depend on *Q* or more specifically on (*QR*) (Volino and Dianoux, 1980). In practice, the summation series in the above equation can be truncated at those values of *n* and *l* for which $A_{n}^{l}$ do not contribute up to *Q*_max_*R*. In the present case, *Q*_max_ = 1.8 Å^−1^ and *R* ~ 3Å, hence *Q*_max_*R* = 5.4. It has been shown (Volino and Dianoux, 1980) that for *QR* ~ 20 (which is sufficiently larger than the present case), one needs to take into account about 100 terms to obtain a numerical accuracy of ~10^−3^. In the present case where *Q*_max_*R* = 5.4 we have used first 100 terms of the summation series.

To estimate the time scale of the internal motion of the lipid, a detailed analysis of the Γ_int_(*Q*) has been carried out. Since no analytical expression exists for Γ_int_(*Q*) within the LTD model, it was numerically calculated using the quasielastic term of Equation (8). D_int_ is obtained from the least-squares fitting of Γ_int_(*Q*) (Figure 7). The *p*_*x*_ and *R* as obtained from the description of EISF have been used in the fitting. As evident from Figure 7, that model could describe the obtained HWHM quite well. The obtained diffusion coefficients *D*_*int*_ for both the systems are given in Table 1. Again a ~ 20% increase in *D*_*int*_ is found at both temperatures suggesting an enhancement of the internal motions in the presence of the IL.

The data obtained here indicates that [DMIM][BF4] does affect the dynamics of the liver membrane, by enhancing both lateral motions within the leaflet and also the localized internal motion of the lipids. A recent molecular dynamics (MD) simulation study (Yoo et al., 2014) has shown that the imidazolium based ILs penetrate within the lipid membrane and have a preferred orientation in the lipid membrane. Alkyl chains prefer to orient parallel to the lipid molecules and the imidazolium group interacts with the head group. This was further supported by Isothermal Titration Calorimetry (ITC) measurements which showed stoichiometry ratios for IL of ~2. Incorporation of IL inside the membrane creates disorder, supported by neutron and x-ray reflectivity measurements (Benedetto et al., 2014; Bhattacharya et al., 2017) which showed that addition of IL leads to membrane thinning.

These results are in contrast with the recent QENS studies on membranes with antimicrobial peptides (AMPs), where it was reported that the addition of cationic antimicrobial peptides in zwitterionic PC (Sharma et al., 2015b, 2016b) as well as in anionic PG (Mitra et al., 2020) restricts the dynamics of the membrane. Moreover, these peptides have shown that the restriction is mainly on the lateral motion of the lipid, while the internal motion of the lipids remained unchanged. Observed differences between the action of ILs and antimicrobial peptides on the membranes can be explained on the basis of their locations within the membrane. Using orientated circular dichroism (OCD), it has been shown that at low concentrations, cationic AMPs bind on the surface of the lipid membrane (Sharma et al., 2016b). These observations were also supported by ITC data which showed that stoichiometry ratio for these AMP is high (~few tens of the lipid), further indicating that these AMPs bind on the surface on the lipid membrane (Mitra et al., 2020). As the lateral motion of the lipid involves diffusion of the whole lipid within the leaflet and the internal motion of the lipid is localized, the AMPs affect mainly the lateral motion of the lipid. Comparing the results with other membrane active molecules such as NSAIDs (Sharma et al., 2019b, 2020), vitamins (Sharma et al., 2016a), cholesterol (Sharma et al., 2015b) etc., it can be concluded that effects of the membrane active molecules are correlated with the location of the molecules in the lipid membrane. If the additive molecules are located on the surface of the membrane it mainly affects the lateral motion of the lipid molecules. However, if molecules penetrate within the lipid membrane, both lateral and internal motions of the lipid are affected. Addition of these molecules enhances or restricts the membrane dynamics depending on their nature. It is worth mentioning that cholesterol also penetrates into the membrane core similar to the IL, but in contrast, it restricts both the lateral and internal motions of the lipid in the fluidic phase of membrane (Sharma et al., 2015b). This can be understood as the incorporation of cholesterol in the membrane's fluidic phase brings more order in the membrane while IL brings disorder in the membrane. These studies reveal that membrane dynamics are highly sensitive to the presence of membrane active molecules and can be used to decipher interactions of these additives with the cell membrane.

## Conclusions

Quasielastic neutron scattering (QENS) has been used to study the effects of an ionic liquid (IL) on the dynamics of the membrane formed by liver extract lipid. A prototype imidazolium based IL [DMIM][BF4], has been used for the present study. QENS data analysis indicates the presence of two distinct lipid motions, (i) lateral motion of the lipid within the leaflet and (ii) internal motions of the lipid, in the ps timescale. The lateral motion of the lipid follows a continuous diffusion whereas the internal motions can be described using localized translational diffusion. Incorporation of IL in the liver lipid membrane is found to enhance the dynamics in the membrane, indicating an increase in membrane fluidity. Both lateral and internal motions of the lipid are accelerated due to the addition of the IL. This is explained on the basis of the location of the IL within the lipid membrane. As the IL goes into the membrane core and adopts a preferred orientation parallel to the lipid, increasing the disorder of the lipids and leading to enhanced mobility. Results are compared with various membrane active bio-molecules such as antimicrobial peptides, cholesterol, drugs, and vitamins. This comparison suggests a strong correlation between the location of the additive inside a lipid membrane and the microscopic dynamics of the lipid membrane. The present study also shows that a more physiologically relevant bio-membrane model system can be used for the study of the interaction of foreign molecules with cellular membranes. In particular, this model system would be an excellent one in which to incorporate trans- and peripheral membrane proteins to figure out if the effects of an ionic liquid on a living organism is related to purely the membrane reorganization or to a possible denaturation of the structure of proteins, or a combination of both.

## Data Availability Statement

The data that support the findings of this study are available from the corresponding author upon reasonable request. The QENS raw data can be found in Dr. Ramaprosad Mukhopadhyay et al. (2017): Dynamics in Bovine Lipid with ionic liquid, STFC ISIS Neutron and Muon Source, https://doi.org/10.5286/ISIS.E.89903473.

## Author Contributions

VS, RM, and SG designed the research project. VS, RM, and VG carried out experiments. VS wrote the manuscript with the inputs from all the authors. All authors contributed to the article and approved the submitted version.

## Conflict of Interest

The authors declare that they have no conflicts of interest with the contents of this article.
